# Automatic lesion detection and segmentation in ^18^F-flutemetamol positron emission tomography images using deep learning

**DOI:** 10.1186/s12938-022-01058-8

**Published:** 2022-12-20

**Authors:** Chan Ju Ryu

**Affiliations:** Department of Nuclear Medicine, Cha University Bundang Medical Center, 59, Yatap-ro, Bundang-gu, Seongnam, Gyeonggi-do 13496 Korea

**Keywords:** Amyloid, ^18^F-flutemetamol, Positron emission tomography (PET), Segmentation, Convolutional neural network (CNN), Alzheimer's disease

## Abstract

**Background:**

Beta amyloid in the brain, which was originally confirmed by post-mortem examinations, can now be confirmed in living patients using amyloid positron emission tomography (PET) tracers, and the accuracy of diagnosis can be improved by beta amyloid plaque confirmation in patients. Amyloid deposition in the brain is often associated with the expression of dementia. Hence, it is important to identify the anatomically and functionally meaningful areas of the human brain cortex surface using PET to diagnose the possibility of developing dementia. In this study, we demonstrated the validity of automated ^18^F-flutemetamol PET lesion detection and segmentation based on a complete 2D U-Net convolutional neural network via masking treatment strategies.

**Methods:**

PET data were first normalized by volume and divided into five amyloid accumulation zones through axial, coronary, and thalamic slices. A single U-Net was trained using a divided dataset for one of these zones. Ground truth segmentations were obtained by manual delineation and thresholding (1.5 × background).

**Results:**

The following intersection over union values were obtained for the various slices in the verification dataset: frontal lobe axial/sagittal: 0.733/0.804; posterior cingulate cortex and precuneus coronal/sagittal: 0.661/0.726; lateral temporal lobe axial/coronal: 0.864/0.892; parietal lobe axial/coronal: 0.542/0.759; and striatum axial/sagittal: 0.679/0.752. The U-Net convolutional neural network architecture allowed fully automated 2D division of the ^18^F-flutemetamol PET brain images of Alzheimer's patients.

**Conclusions:**

As dementia should be tested and evaluated in various ways, there is a need for artificial intelligence programs. This study can serve as a reference for future studies using auxiliary roles and research in Alzheimer's diagnosis.

## Background

Dementia is one of the neurodegenerative diseases and can be classified into dementia, vascular dementia, and treatable dementia based on the cause. Pathological findings of neurodegenerative diseases indicate the accumulation of amyloids in the brain as one of the primary causes. Of these, abnormal clustering of beta amyloid (Aβ) in the brain can damage the nerve cells [1–3]. The development of molecular imaging techniques has greatly influenced the pathological physiological diagnosis and research of Alzheimer's disease (AD); in particular, amyloid (A) imaging can be used as a neurodegenerative biomarker to assess the presence and extent of Aβ deposition in vivo during AD diagnosis [4, 5].

In amyloid positron emission tomography (PET), an image biomarker along with a radioactive isotope is injected to bind to the amyloid protein. In early biomarker studies, carbon radioisotope (^11^C) was mainly used, but in recent times, fluorine isotope (^18^F) is used as it has a longer half-life [6, 7]. Amyvid (florbetapir, 2012) was originally approved by the US Food and Drug Administration for Aβ target imaging; however, Vizamyl (flutemetamol, 2013) and NeuraCeq (florbetaben, 2014) have also been approved of late for the same purpose [8].

Currently, the standard for determining Aβ deposits under clinical settings involves careful visual assessment by skilled doctors [9]. However, the classification accuracy of the disease type depends on the training and experience of the doctor; further, burnout problems with doctors may result in misdiagnosis of medical readings, and in the case of low levels of Aβ deposition, visual evaluations may be difficult [10]. There are different types of radiation tracers that can be used with PET to detect A markers, which are characteristic of Alzheimer's neuropathology, and visual readings of the ^18^F-flutemetamol scans used in studies were often obtained according to a color scale (Sokoloff, Rainbow, or Spectrum) [11–13]. These color scales require accurate depictions of the cortex and reference regions compared to regular gray-level scales and are influenced by the PET machines and reconstruction algorithms, which render the standardized criteria for positive A identification difficult and may limit accurate diagnosis by a doctor’s visual assessment [14].

The main detection model using convolutional neural network (CNN), i.e. semantic segmentation, can distinguish the meaningful parts of an image or video by image processing [15, 16]. Using the 18F-FET PET image proposed by Blanc-Durand et al. [17], glioma was automatically detected and segmented using a full 2D U-Net CNN. In addition, Falk et al. [18] proposed a segmentation study for cell counting, detection, and morphological measurement using the U-Net algorithm.

Our study is important for determining whether ^18^F-flutemetamol PET imaging can be used to effectively distinguish the characteristics of Aß deposition from the diagnostic group on an unstandardized color scale. We extracted patient data and standard imaging of deep learning images through preprocessing (image editing) of ^18^F-flutemetamol PET images, which are not standardized with the image editing program (MIM) used in hospitals and constructed an image database which we used to analyze data from patients regarding similar shapes and positions of the brain. Then, an additional mask branch was inserted to predict whether each pixel corresponded to an object, and a binary mask was obtained to determine whether each pixel in a boundary box was part of the object. The primary purpose of this study was to develop, learn, verify, and test 2D-CNNs to classify negative (i.e. no amyloid accumulation) and positive (i.e. amyloid accumulation) ^18^F-flutemetamol scans by training deep learning (DL) networks using U-Net structures [19, 20].

## Results

The classification learning performance of the U-Net model using the ^18^F-flutemetamol images was confirmed for 440 images, of which 264 were positive and 176 were negative. The learning results were compared based on six parameters: mean intersection over union (IoU), accuracy, specificity, sensitivity, precision, and F1-score. The ground true images and the images form the results were compared pixel-wise, and the TP, FP, FN, and TN values were represented by a confusion matrix; then, the six parameters were calculated using Eqs. (6)–(11). Table 1 summarizes the overall segmentation performance of the CNN architecture.

**Table 1:** Machine learning per-segment similarity coefficient and segmentation performance results

Segment	Frontal lobe	PCC and Precuneus	Lateral temporal lobe	Parietal lobe	Striatum
Mean IOU	0.804	0.726	0.892	0.759	0.752
Accuracy (%)	90.3	92.0	97.6	97.3	96.7
Specificity (%)	81.4	88.0	96.0	100.0	100.0
Sensitivity (%)	100.0	100.0	100.0	100.0	94.1
Precision (%)	85.9	90.8	95.2	100.0	100.0
F1-score (%)	91.6	93.2	97.5	95.9	94.0

*IoU* intersection over union, *PCC* posterior cingulate cortex

**Fig. 1:**
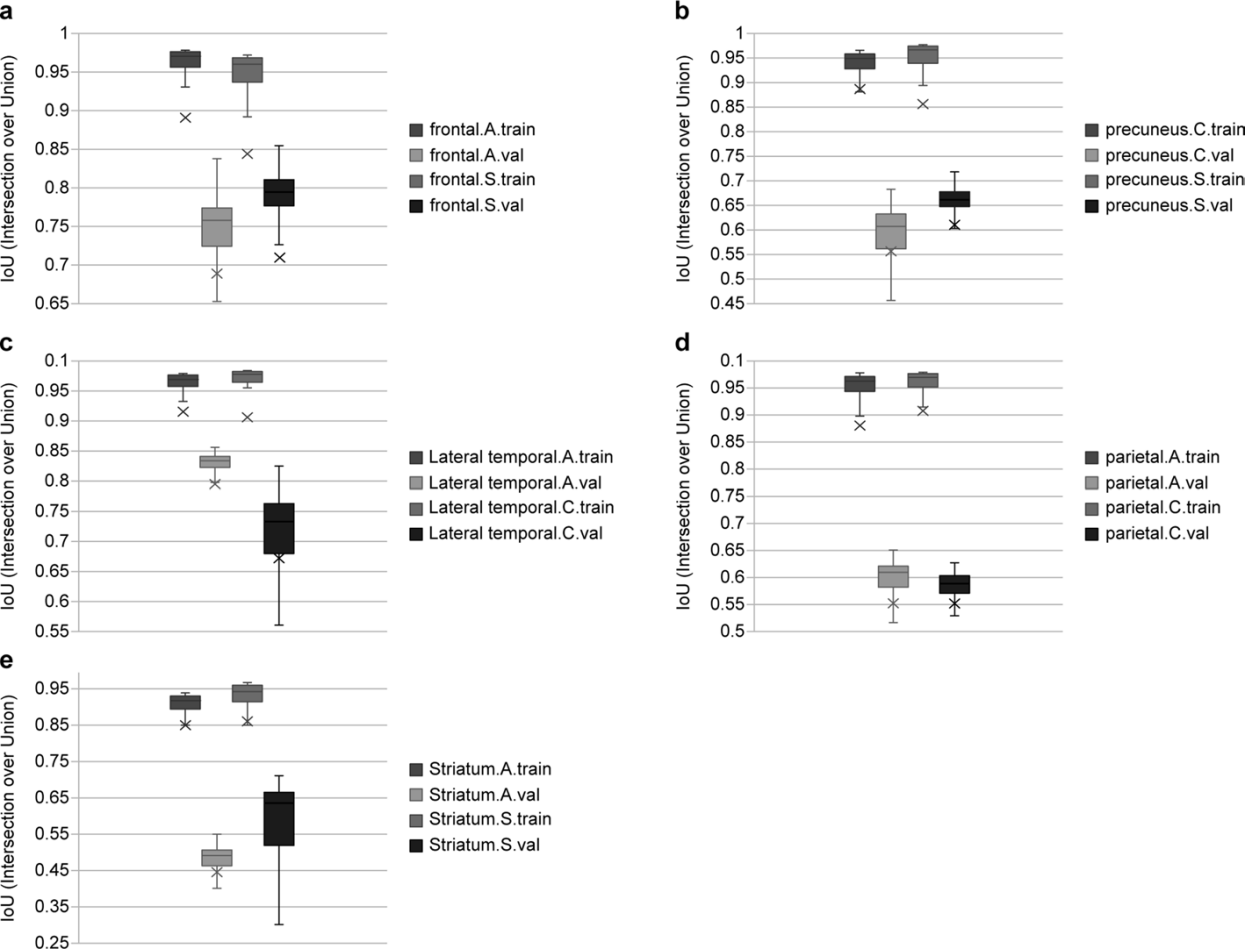
Machine learning per-segment similarity coefficient and segmentation performance: **a** Frontal lobe, **b** PCC and precuneus, **c** Lateral temporal lobe, **d** Parietal lobe, **e** Striatum

Figure 1 shows the mean, median, and maximum IoU for the training and validation datasets, depending on the axial directions of the five target zones. The proposed network has average, median, and maximum IoU segmentation scores for Alzheimer’s lesions, which were 0.689, 0.757, and 0.840 in the axial direction in the frontal lobe and 0.709, 0.795, and 0.860 in the sagittal direction. In the posterior cingulate cortex (PCC) and precuneus zones, these values were 0.556, 0.607, and 0.680 in the coronal direction and 0.610, 0.662, and 0.790 in the sagittal direction. In the lateral temporal lobe, the values were 0.759, 0.833, and 0.850 in the axial direction and 0.671, 0.732, and 0.820 in the coronal direction. In the parietal lobe, the values were 0.655, 0.611, and 0.670 in the axial direction and 0.655, 0.609, and 0.683 in the coronal direction. In the striatum, the values were 0.534, 0.552, and 0.550 in the axial direction and 0.543, 0.632, and 0.710 in the sagittal direction.

**Fig. 2:**
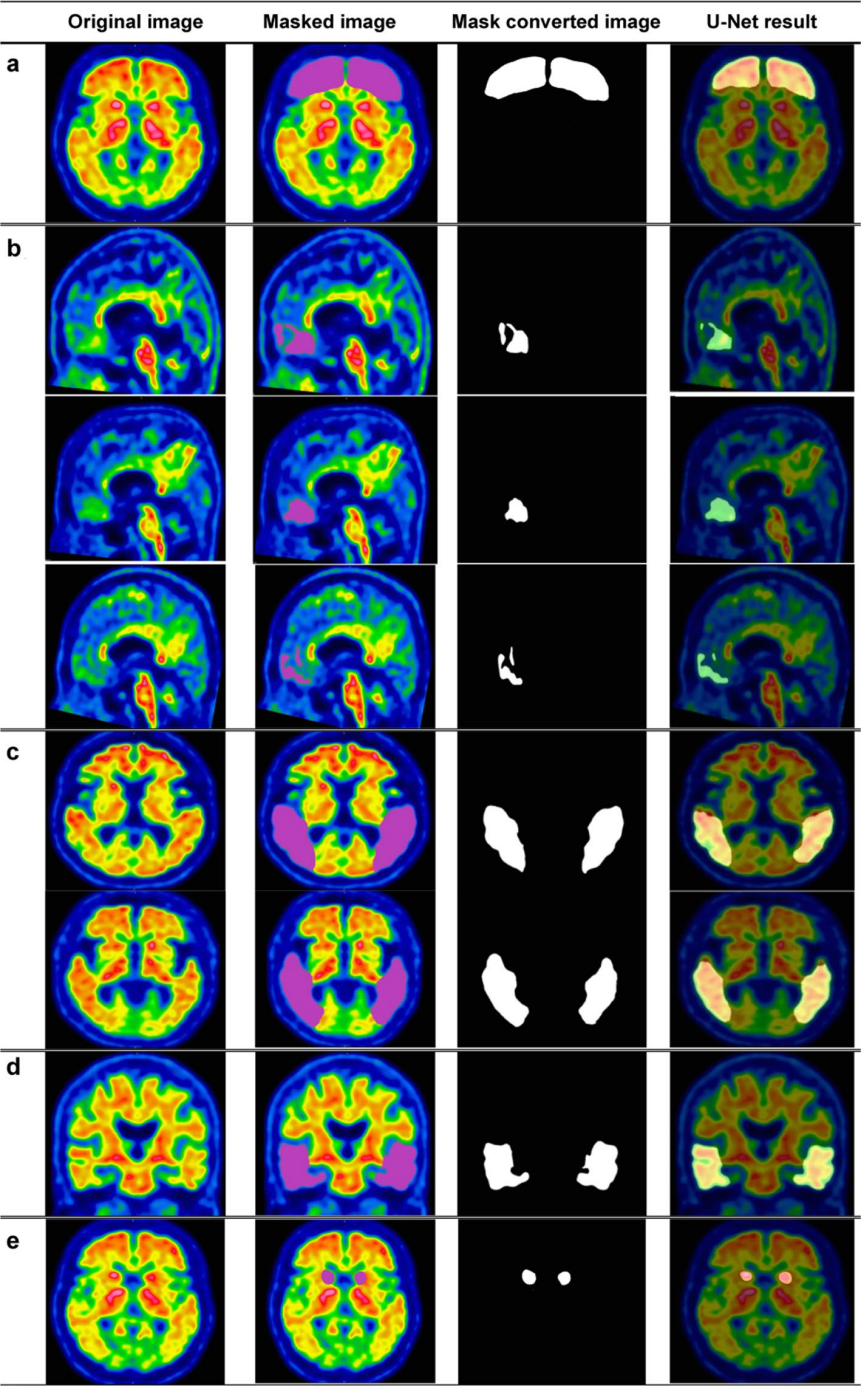
Positive image: **a** Frontal lobe (axial) region, **b** frontal lobe (sagittal) region, **c** lateral temporal lobe (axial) region, **d** Lateral temporal lobe (coronal) region, **e** Striatum (axial) region

**Fig. 3:**
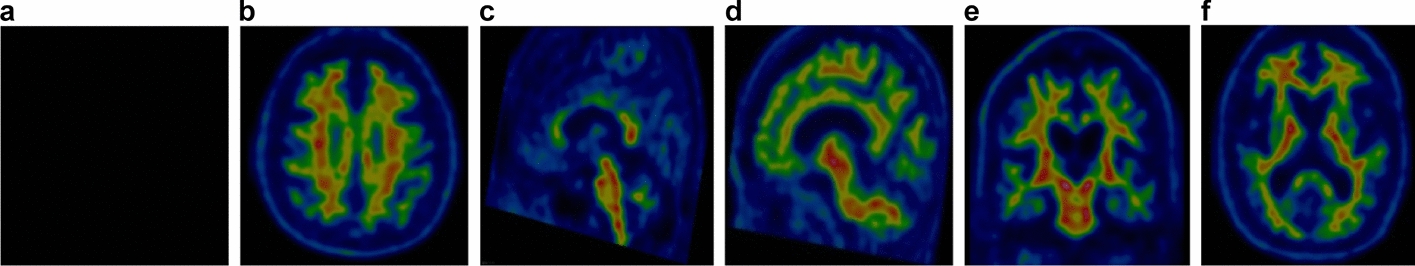
Negative image: **a** mask converted image, **b** parietal lobe (axial) region segmentation image, **c** frontal lobe (sagittal) region segmentation image, **d** striatum (sagittal) region segmentation image, **e** lateral temporal lobe (coronal) region segmentation image, and **f** frontal lobe (axial) region segmentation image

Radiologists were recruited to subjectively evaluate these segmentation results, with the resulting images being split into the five amyloid zones, as shown in Figs. 2 and 3. These results show almost perfect pixel-wise segmentation of the lesions in relation to the positive and negative areas. The subjective evaluations by radiologists revealed that these segmentation results matched the ground truth images. Figure 2 shows an example of a positive image (amyloidosis), with (a) being an original image from a set of amyloid PET data and (b) representing the label of the corresponding amyloid region. Figure 2c shows the outputs of the image using the binary technique; Fig. 2d shows the divided images overlapped with the original images from semantic segmentation.

We measured the operation time for the segmentation using the timer of the graphics processing unit (GPU) to compare the improved performance time for parallel computing using CUDA. Each of the 76 amyloid PET images was tested on the GPU by random selection from the dataset. The test time for the results is the total required time, and the training time is the time required for 2000 training cycles, as shown in Table 2.

**Table 2:** Computing time comparison for the five areas

Computing practice	Equipment	Frontal lobe	PCC and Precuneus	Lateral temporal lobe	Parietal lobe	Striatum
Average test time/s	GPU	10.72	16.35	9.75	8.19	6.87
Average training time/min	GPU	6.3	7.1	9.6	7.2	5.7

*GPU* graphic processing unit, *PCC* posterior cingulate cortex

**Fig. 4:**
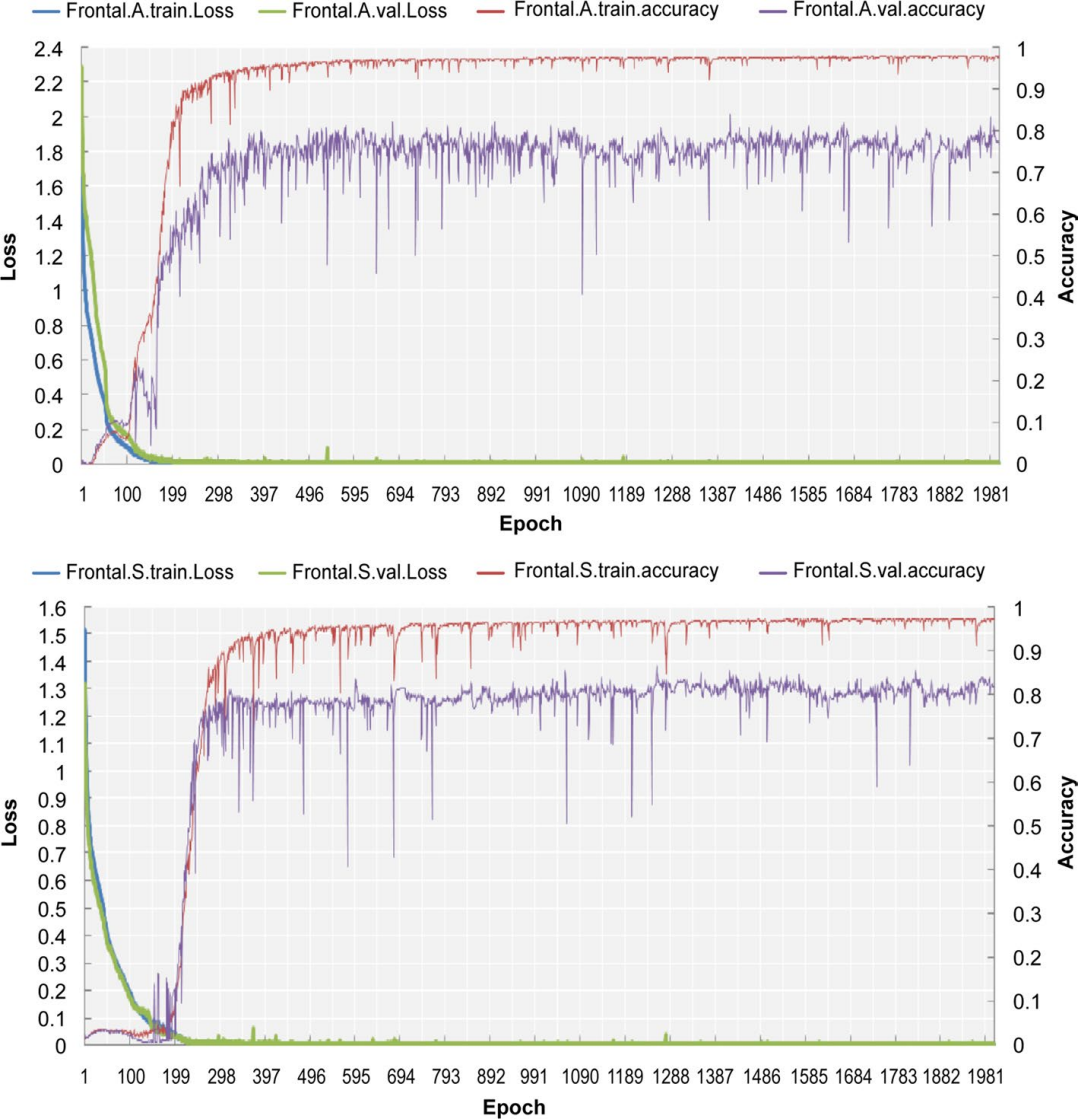
Learning curves for training and validation data for the frontal lobe region

The graphics training performance for the proposed AD lesion segmentation is shown in Fig. 4, with the accuracy and loss curves for the training and validation phases of the network being provided in Table 3. In addition, the U-Net model in Fig. 4 shows fast convergence without overfitting the training data, especially considering that the validation loss is similar to the training loss.

**Table 3:** Loss curves for the training and validation phases of the network

Performance metrics	Frontal lobe	PCC and precuneus	Lateral temporal lobe	Parietal lobe	Striatum
Training loss	0.02816	0.01238	0.02009	0.01909	0.00905
Validation loss	0.00432	0.00168	0.00314	0.00345	0.00147

*PCC* posterior cingulate cortex

## Discussion

The purpose of this study was to implement and evaluate a 2D U-Net CNN for positive and negative area segmentation of ^18^F-flutemetamol amyloid images. The U-Net model used for learning positive and negative segmentations of ^18^F-flutemetamol amyloid images [19] is a neural network model that is optimized for medical image segmentation. Because this network does not use a fully connected layer, the learning speed is high. By attaching and using the neural network used in the max pooling process, the loss of spatial information by dimension reduction is prevented. This has the advantage of producing high-performance segmented results with a small amount of data. According to Lindström et al. [21], PET images were studied to understand the uptake value ratio (SUVR) values and quantitative measurements via reconstruction. In particular, we confirmed that the relative absorption differences in the images increase or decrease due to reconstructions from images of patients with neurodegenerative diseases in the database. Hence, it was concluded that the images acquired through reconstruction required normalization; if a normalized image is not set when dividing deep learning images from medical image data, problems, such as incorrect subdivision by the algorithm and difficulty in obtaining accurate subdivision results, are observed. The visual geometry group (VGG) model is a simple CNN architecture that uses fewer hyperparameters. Deep learning through ^18^F-florbetaben amyloid PET images using VGG [22, 23] and deep learning with 2D CNN-based AD/CN classifications from ^18^F-flortaucipir PET images have also been reported. In the framework development study, since grayscale (0,1) PET image data are used, normalization of the images in the database is not required. However, as in this study, if the ^18^F-flutemetamol amyloid PET images are in color (0–100%), then the images require normalization of the pixel values for the entire brain based on the values of the cerebellar gray matter (or pons). Despite the heterogeneity of the dataset used in the image reconstruction, image quality, pixel concentration based on color scale, and voxel size, the results of the amyloid region segmentation learning showed an IoU value of 0.804 for the frontal lobe, 0.726 for the posterior cingulate cortex, 0.720 for the anterior dental coronary/thalamus, 0.892 and 0.752 for the vertical lobe axis/coronary, and 0.752 for striatum regions, and failure cases (IoU < 0.5) were not recorded. This shows the advantage of generating high-performance segmented results with a small amount of data. The data presented herein highlight the typical high accuracy and low operation time for artificial intelligence (AI) integration through encouraging results for application in the radiation field, especially in the field of nuclear medicine to evaluate dementia.

## Conclusions

In this paper, we propose an in-depth supervisory 2D U-Net model for Alzheimer's lesion segmentation in three-axial (axial, coronal, and sagittal) ^18^F-flutemetamol PET images. The proposed network efficiently segments the amyloid-positive and -negative regions with a mean IoU score of 0.787. The experimental results showed that the proposed U-Net-based algorithm achieved IoU values of 0.733/0.804 in the frontal lobe axial/sagittal, 0.661/0.726 in the PCC and precuneus coronal/sagittal, 0.864/0.892 in the lateral temporal lobe axial/coronal, 0.542/0.759 in the parietal lobe axial/coronal, and 0.679/0.752 in the striatum axial/sagittal slices for the verification dataset. Existing amyloid PET images that are evaluated using deep learning methods are generally grayscale images [24], but our work uses colored images; thus, to the best of our knowledge, for the first time, a deep learning method for efficient segmentation by normalization of the color pixels in the image has been proposed. If additional learning can be performed in the future by collecting data acquired from various scan techniques, it is expected that a computer-assisted diagnostic system can be developed to generate more accurate segmentation results that clearly delineate the positive and negative areas, possibly assisting clinical physicians with insufficient experience to diagnose and treat AD.

## Methods

**Fig. 5:**
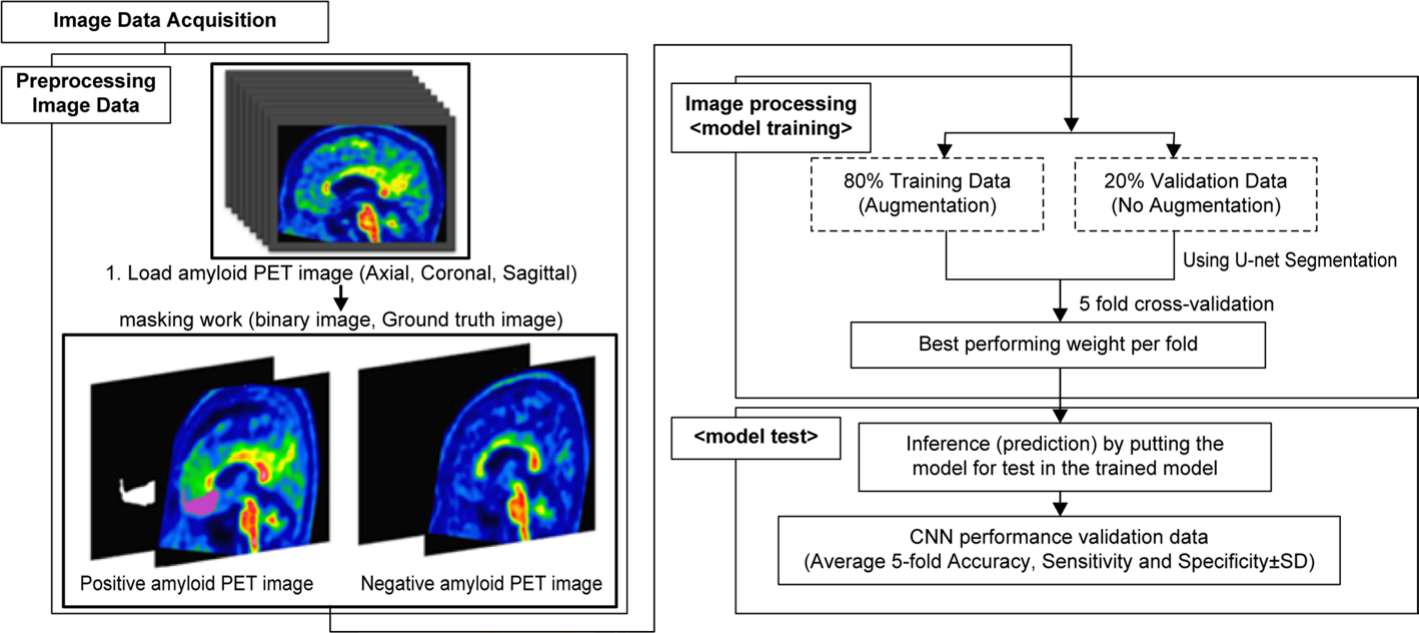
Overall flowchart of the proposed Alzheimer’s disease-positive region segmentation algorithm

The algorithm proposed in this paper comprises four steps: data acquisition, data preprocessing, U-Net training, and U-Net testing. The flowchart for the algorithm is shown in Fig. 5.

### Participants

The institutional review boards of medical center approved this study (IRB No. 2020-02.017) and exempted the need for individual informed consent. This research study was performed in accordance with the principles of the Helsinki Declaration as revised in 2013. We retrospectively collected flutemetamol PET images that were obtained between 06/09/2017 and 05/09/2020 at the medial cancer. An experienced nuclear medicine physicians who received electronic training for vizamyl analyzed the flutemetamol PET images. Considering the formal report as the gold standard, 176 cases were considered as negative for amyloid, and 264 cases were positive for amyloid. For positive cases, we scored each of five domains, namely, frontal lobes, posterior cingulate and precuneus, lateral temporal lobes, inferolateral parietal lobes, and striatum, as positive or negative according to the formal report.

### PET/CT data acquisition

The PET data were acquired using a Biograph mCT Series PET/CT scanner (Siemens Healthcare, Europe); the patient head movements were minimized using a head holder. A low-dose computed tomography (CT) scan was first acquired for attenuation and scatter correction. Then, about 330 ± 30 MBq of the amyloid radiotracer ^18^F-flutemetamol (Vizamyl™; GE Healthcare, Little Chalfont, England, UK) was injected intravenously, and PET was acquired 90–110 min post-injection. The list-mode PET data were reconstructed using the time-of-flight ordered-subsets expectation–maximization and TrueX + time-of-flight algorithm with four iterations and 21 subsets, thus accounting for random, scatter, dead time, and attenuation. A 3.0 mm full width at half maximum post-reconstruction Gaussian filter was used on all PET images. The reconstructed images had a matrix size of 256 × 256 × 200 and a voxel size of 2.0 mm in the three primary directions.

### Image preprocessing

#### Ground truth ^18^F-flutemetamol PET segmentation

**Fig. 6:**
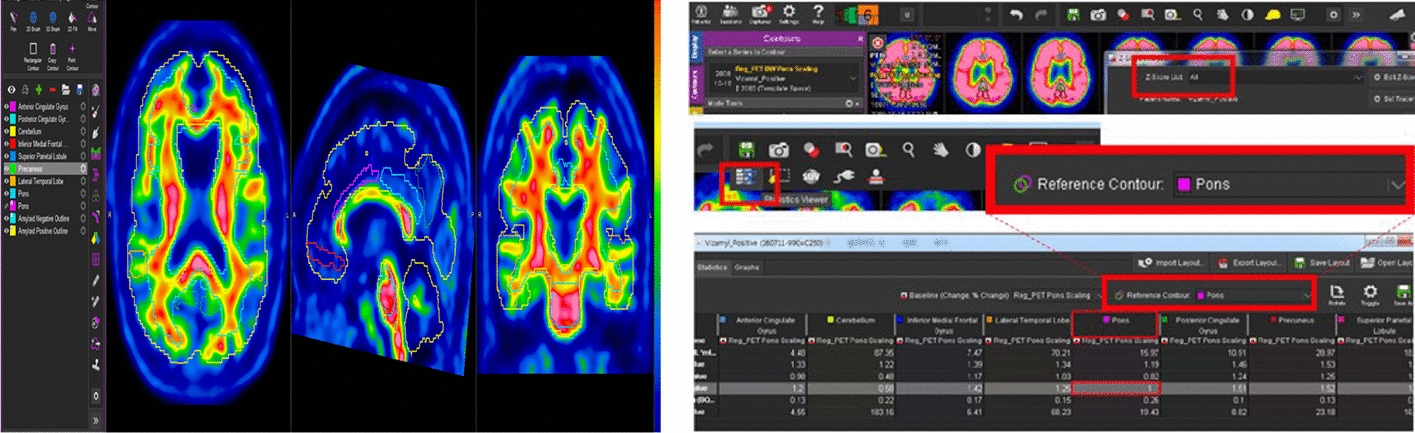
Automatic normalization of the contrast in the five identified areas using MIM software and based on the Pons as the reference

To perform semi-automatic contouring, masks were drawn manually around the white or gray matter of images from five regions (frontal lobe, (PCC) and precuneus, lateral temporal lobe, inferolateral parietal lobe, and striatum) using MIM software (https://www.mimsoftware.com, CEA, version 6.8.6) [12, 25], as shown in Fig. 6. After normalizing the structural position of the patient, each area was detected automatically, and the SUV and *Z*-score values for each area were calculated. Using these two values, the contrast of the image pixels was normalized automatically. Using the pons as a reference, the densities of all areas having white matter were adjusted using the statics viewer, with the pons reference contour ratio of 1 as the standard.

#### Preprocessing

**Fig. 7:**
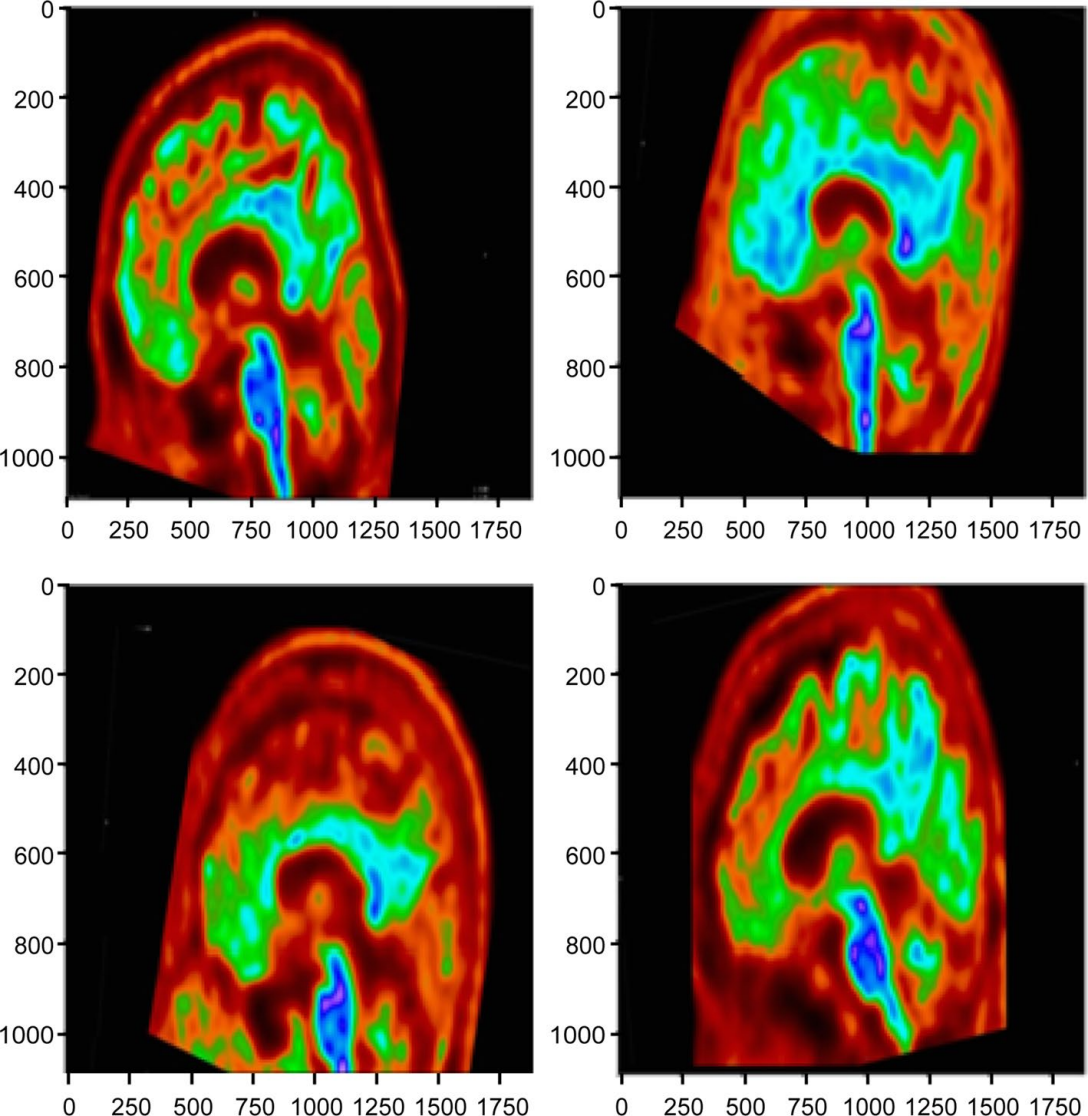
Sample image and its random data augmentation procedures using OpenCV

For computational purposes, all images (masks and summation images) were resized using linear interpolation to a volume of 256 × 256 × 159 mm^3^ voxels. Each volume was normalized to the mean and standard deviation values of all images. To avoid overfitting, a data augmentation strategy was used to enlarge the training dataset. As shown in Fig. 7, this procedure included rotations (− 10° and + 10°), *X*- and *Y*-axis translations (− 0.1, 0.1), and shearing (− 10°, + 10°). To evaluate the segmentation procedure under clinical conditions, the dataset was randomly split between a training set and a validation set, constituting 80% and 20% of the entire image dataset, respectively.

#### Loss function

The five zones identified account for only a small proportion of the total brain volume. In this study, the IoU loss [26] expressed by Eq. (1) was used to segment the amyloid positive area, and its value ranges from 0 to 1.

1 $${L}_{\mathrm{IoU}}=1-\frac{\sum_{i}^{N}{P}_{i}{g}_{i}}{\sum_{i}^{N}{P}_{i}+\sum_{i}^{N}{g}_{i}-\sum_{i}^{N}{P}_{i}{g}_{i}},$$

where ${p}_{i}$ represents the softmax value of the *i*th voxel output by the segmentation subnetwork, as shown in Eq. (2); ${g}_{i}$ represents the value of the $i$th voxel in the positive area gold-standard mask. If a voxel belongs to the target region (hippocampus), then its value is 1; otherwise, its value is 0 [27].

2 $${p}_{i}=\frac{{\mathrm{e}}^{{y}_{i}}}{\sum_{i=1}^{N}{e}^{{y}_{i}}}.$$

The cross-entropy loss function [28] expressed by Eq. (3) was used to classify the pathological state diagnosis of the brain:

3 $${L}_{\mathrm{cross-entropy}}=-\sum_{k}{t}_{k}\mathrm{log}{y}_{k},$$

where ${t}_{k}$ is the correct solution, such that its index is 1 when correct, and 0 otherwise. ${y}_{k}$ is the output of the softmax activation function and is calculated using Eq. (4).

4 $${y}_{k}=\frac{{\mathrm{e}}^{{y}_{k}}}{\sum_{k=1}^{2}{e}^{{y}_{k}}}.$$

#### Implementation

We trained the model using a workstation with an NVidia GeForce GTX 1080 GPU (8 GB), Ubuntu 14.04 operating system, and CUDA 11.0, by employing the deep learning toolkit Python (v.3.7.6) [29, 30]. The networks were trained using the stochastic gradient descent Adam optimizer method [31], with the IoU as an accuracy measure for the segmentation procedure, and negative IoU as the loss function that is backpropagated through the CNN [32, 33]. The batch size was set to four. The learning rate was set to ${10}^{-5}$ initially, and the model was trained for up to 2000 epochs.

#### Architecture of the U-Net

**Fig. 8:**
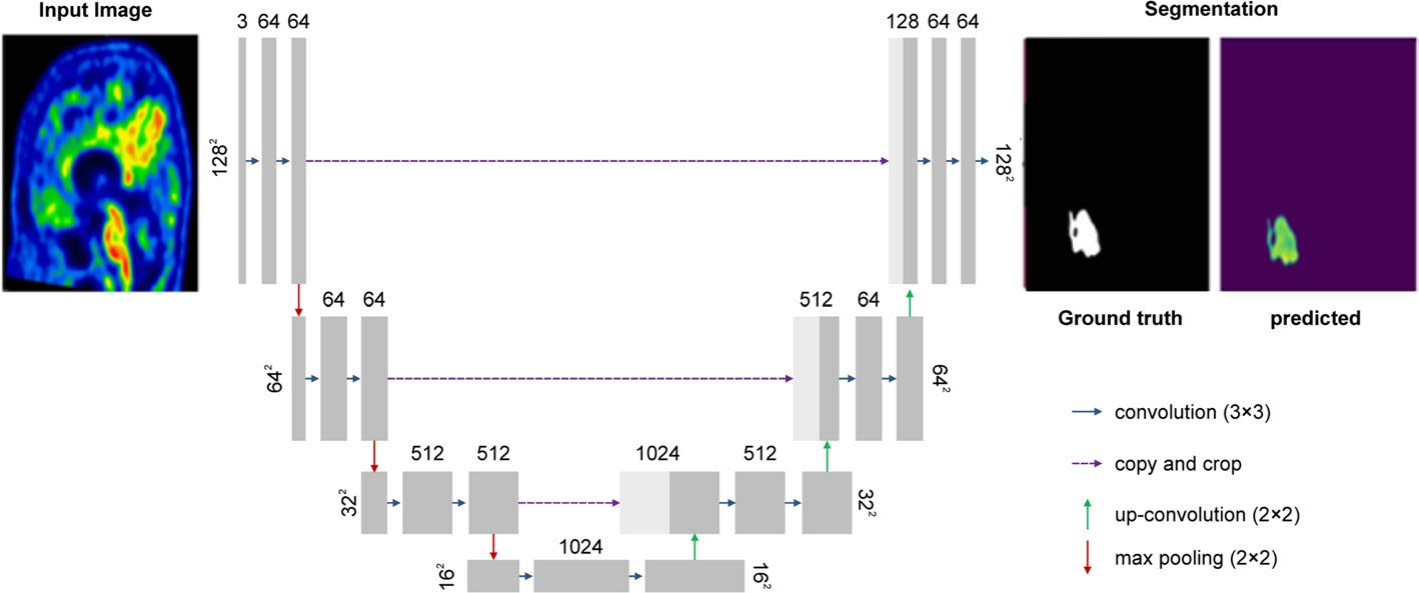
U-Net model

As shown in Fig. 8, U-Net is a popular end-to-end encoder–decoder network for semantic segmentation that was originally formulated for biomedical image segmentation tasks [34]. The U-Net developed by Christ et al. [34] extended the fully convolutional networks [35] with a U-shaped architecture, which allowed features from the shallower layers to combine with those from the deeper layers. U-Net consists of a contracting path to capture the features and an asymmetric expanding path that enables precise localization and segmentation of the pixels. This architecture is U-shaped and skipping connections join the high-resolution features from the contracting path to the upsampled outputs of the expanding path.

After collecting the required features in the encoding path, the decoding path performs nonlinear upsampling of the feature maps before merging with the skipped connections from the encoding path [36], followed by two 3 × 3 convolutions, and each followed by an element-wise rectified linear unit. The skip concatenation allows the decoder to learn at each stage the relevant features that are lost when pooled in the encoder. In the final layer, a 1 × 1 × 1 convolution is used to map each component feature vector to the desired number of classes (two in our study) [37].

In total, the network had 11 convolutional layers and 214,748 parameters to be trained. The predicted mask from the U-Net was followed by a morphological dilation operation with 3 × 3 × 3 square connectivity. Fixed thresholding was performed, where the threshold was set to 1.3 times the mean value of a hemispheric swap of the predicted U-Net mask to match the procedure performed for the ground truth. All computations were performed using Python 3.7.6 with NumPy, TensorFlow 2.1 [38]. U-Net required a training time of approximately 9.5 h on the NVidia GeForce GTX 1080 GPU (8 GB).

#### Evaluation criteria

In this study, we chose the IoU measure, which is also known as the Jaccard index, to evaluate the positive area segmentation performance. The IoU measure quantifies the ratio of overlap between the ground truth mask and segmented image [39] and its mathematical definition is given in Eq. (5), which was obtained by subtracting the dice loss value from 1:

5 $${L}_{\mathrm{IoU}}=\frac{\sum_{i}^{N}{P}_{i}{g}_{i}}{\sum_{i}^{N}{P}_{i}+\sum_{i}^{N}{g}_{i}-\sum_{i}^{N}{P}_{i}{g}_{i}}.$$

The meaning of the parameters in Eq. (5) is the same as that in Eq. (1).

The brain pathological state diagnosis performance was evaluated based on the accuracy, specificity, sensitivity, precision, and F1-score. The formulas for these indicators are as given in Eqs. (6)–(10).

6 $$\mathrm{Accuracy}=\frac{\mathrm{TP }+\mathrm{ TN}}{\mathrm{TP }+\mathrm{ TN }+\mathrm{ FP }+\mathrm{ FN}},$$

7 $$\mathrm{Specificity}=\frac{\mathrm{TN}}{\mathrm{TN }+\mathrm{ FP}},$$

8 $$\mathrm{Sensitivity}=\frac{\mathrm{TP}}{\mathrm{TP }+\mathrm{ FN}},$$

9 $$\mathrm{Precision}=\frac{\mathrm{TP}}{\mathrm{TP }+\mathrm{ FP}},$$

where TP, TN, FP, and FN represent the true-positive, true-negative, false-positive, and false-negative numbers, respectively.

10 $$\mathrm{F}1-\mathrm{score }=\frac{2 *\mathrm{ Precision }*\mathrm{ Sensitivity}}{\mathrm{Precision }+\mathrm{ Sensitivity}}.$$

## Data Availability

The datasets generated and/or analyzed during the current study are not publicly available due to security of research data concerns but are available from the corresponding author on reasonable request.

## Abbreviations

PET: Positron emission tomography
^18^F-FMM: 18F-flutemetamol
CNN: Convolutional neural network
IoU: Intersection over union
